# Densities, Viscosities, and Self-Diffusion Coefficients of Octan-1-ol and Related Ether-Alcohols

**DOI:** 10.1021/acs.jced.4c00195

**Published:** 2024-07-03

**Authors:** Markus M. Hoffmann, Anthony A. Gonzalez, Mandy T. Huynh, Kashane K. Miller, Torsten Gutmann, Gerd Buntkowsky

**Affiliations:** † Department of Chemistry and Biochemistry, State University of New York Brockport, Brockport, New York 14420, United States; ‡ Institute of Physical Chemistry, Technical University Darmstadt, Peter-Grünberg-Straße 8, D-64287 Darmstadt, Germany

## Abstract

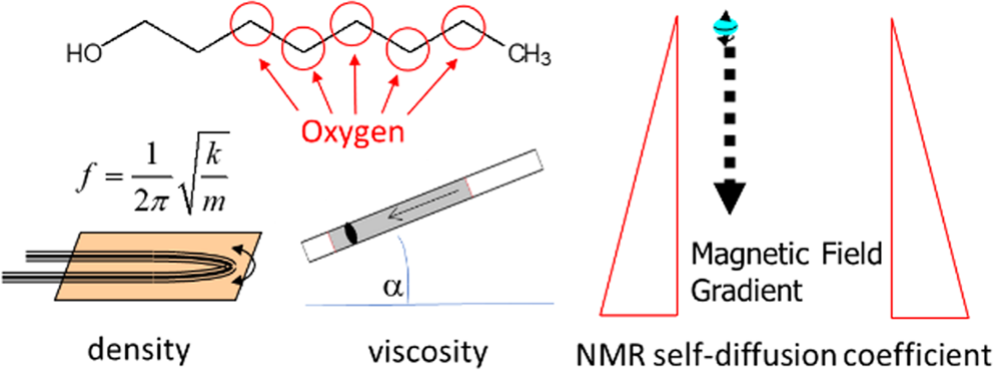

Density, viscosity, and self-diffusion coefficients are reported for octan-1-ol and the related ether-alcohols 2-pentoxy-ethan-1-ol, 3-butoxypropan-1-ol, 4-propoxybutan-1-ol, 5-ethoxypentan-1-ol, and 6-methoxyhexan-1-ol covering temperature ranges from 298.15 to 359.15 K. These new data reveal structure–property relationships affected by the presence and the position of the ether moiety in the molecular structure of the ether-alcohols. Compared to octan-1-ol, the presence of the ether moiety causes an increase in intermolecular hydrogen bonding interactions, resulting in higher densities. The increase in density is less pronounced for those ether-octanols that engage in intramolecular hydrogen bonding. As for the effects of the ether moiety on the dynamics, these are generally faster for the ether-alcohols compared to octan-1-ol, suggesting that hydrogen bonding between ether oxygen and hydroxy hydrogen is weaker compared to hydrogen bonding between two hydroxy groups. The activation energies obtained from an Arrhenius analysis are higher for translational motion than for momentum transfer for all alcohols. There are additional finer details across the ether alcohols for these activation barriers. These differences cancel out for the mathematical product of self-diffusion coefficient and viscosity (*Dη*). The effect of water impurities on the studied properties was also investigated and found to lead to small increases in densities for all alcohols. Viscosities decrease for octan-1-ol and 2-pentoxyethan-1-ol but increase for the other ether-alcohols that can engage in intramolecular hydrogen bonding.

## 1 Introduction

The motivation for studying the properties of octan-1-ol and related ether-alcohols is derived from the desire to better understand the hydrogen bonding interactions of polyethylene glycol (H–[O–CH_2_–CH_2_]_n_–OH), PEG. PEG is an inexpensive, environmentally friendly chemical based on its nontoxicity, low vapor pressure, and biodegradability.^1,2^ PEGs of molar weight less than 1000 g·mol^–1^ are liquid at 298.15 K and have been successfully used as environmentally benign solvent media in chemical synthesis, as recent reviews demonstrate.^3−5^

PEGs are sold as polydisperse mixtures where the product name, such as PEG200, includes the number value of the approximate average molar mass (200 g·mol^–1^ in this case). A recent molecular dynamics (MD) study showed that hydrogen bonding interactions are quite complex in PEG200 because of the multitude of possibilities for inter- and intramolecular hydrogen bonding interactions between the hydroxy as well as the ether functionalities of the ethylene glycol oligomers in PEG200.^6^ A peculiar propensity for intramolecular hydrogen bonding was observed for tetraethylene glycol and, to a smaller extent, also for triethylene glycol. Furthermore, adjustments made to the used force field to better reproduce experimental data of density, viscosity, and self-diffusion coefficients lead to an overall reduction in hydrogen bonding interactions with a concurrent shift toward intramolecular hydrogen bonding.

In this study, we set out to better understand the interplay of intramolecular and intermolecular hydrogen bonds in PEG by investigating the properties of density, viscosity, and self-diffusion coefficients for a series of octan-1-ol related ether-alcohols, which have a related but overall simpler molecular structure. Specifically, these ether-alcohols possess only one hydroxy and one ether functional group, where the position of the ether functional group relative to the hydroxy group varies systematically (see Table 1 for chemical structures). Thus, there is only one intramolecular hydrogen bonding interaction possible in these ether-alcohols, namely, between the hydroxy hydrogen and the ether oxygen. This study also includes 1-octanol, which does not possess an ether functionality and thus serves as a reference compound to understand the structure–property relationships introduced by the presence of the ether functionality. Furthermore, as we will show, there are hardly any physical property data available in the literature for the ether-alcohols in contrast to octan-1-ol, which thus allows verification of measurement accuracy by comparison with available literature data. However, even for octan-1-ol, the available literature data is limited, and this study covers temperatures, for which presently presently only 1-2 datasets exist in the literature on density, viscosity, and self-diffusion, to the best of our knowledge. Thus, the new physical property data should be of value for research involving octan-1-ol. Octan-1-ol is well-known as an amphiphile and may in fact be viewed as the E_0_C_8_ member of the E_m_C_n_ type surfactants where E is an ethylene oxide unit. As such, octan-1-ol is a relevant molecule for studies involving nonionic surfactants. For example, octan-1-ol is used as a model compound to mimic membranes.^7,8^ It is also a standard to determine distribution coefficients of a solute of interest between octan-1-ol and water to assess its hydro/lipophilicity.^9^

**Table 1:**
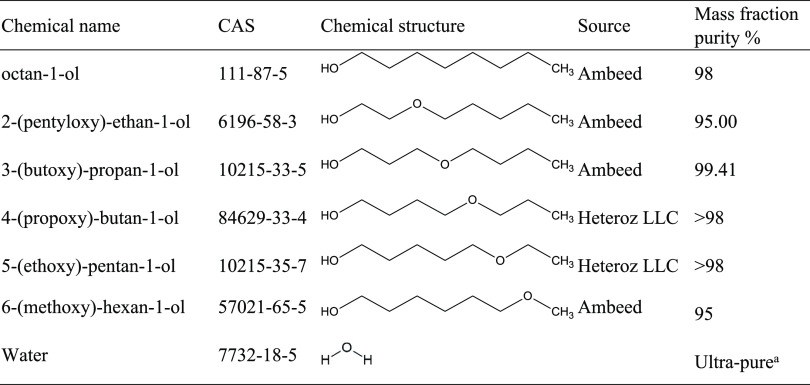
Information on the Chemicals Used

a As defined by electrical resistance, which was 18.18 MΩ.

The organization of the remainder of the report is as follows. After the description of the sample preparation and measurement details in Section 2, Section 3 summarizes the obtained experimental data and the immediate observable trends. Section 4 begins with a brief examination of the data quality and then moves to a molecular-level interpretation of the experimental results. The discussion focuses on how the position of the ether functional group in the molecular structure impacts the measured physical properties as well as derived quantities calculated from the application of the Arrhenius and the Stokes–Einstein relation to the measured viscosity and self-diffusion coefficients. The observed trends obtained from these comparisons are explained within the context of inter- and intramolecular hydrogen bonding. Finally, Section 5 summarizes the main insights obtained from the careful comparisons between the studied alcohols and provides an outlook for future work.

## 2 Experimental Section

### 2.1 Preparation of Samples

Specifications of the investigated chemicals are listed in Table 1. No further purification was attempted. The chemicals were stored, and samples were prepared under nitrogen gas in a Vigor Gas Purification Technologies glovebox. The samples were generally not exposed to the atmosphere during measurements and were measured the same day. After measurement of the as-received chemicals, small amounts of water were added to check the effect of water, the most common sample impurity, on the physical properties. Samples were shaken vigorously for several minutes to ensure sample uniformity. The water content of each sample was measured after density and viscosity measurements were completed using a Mettler Toledo fritless C20 Coulometric Karl Fischer titrator, where the mass of the added sample was determined using a Mettler Toledo model AG104 balance with 0.1 mg precision.

NMR samples were prepared as follows. A melting tube capillary was filled with a sample to about 3/4 of its length by means of a 1 mL plastic syringe with a stainless-steel gauge-20 blunt needle. The capillary was flame-sealed immediately after removal from the glovebox. The sealed capillary was then placed into a standard 5 mm NMR tube, and lock solvent, typically DMSO-*d*_6_, was added. The NMR tube was capped with a standard NMR tube plug, which was then wrapped with parafilm.

### 2.2 Density

Densities were measured from 298.15 to 358.15 K by an Anton Paar, model DMA 4100 oscillating tube density meter. The instrument controls the temperature with an accuracy of 0.02 K and applies a sample viscosity correction to the measured densities. The density of pure water agreed with literature values within 0.0001 g·mL^–1^. Density measurements were repeated three times, and the results also agreed within 0.0001 g·mL^–1^. Therefore, density measurement uncertainty was not limited by the instrument but by the purity of the sample (see Table 1), and appropriate standard uncertainties are provided in Tables 2–7. However, the effect of water as an impurity was investigated as presented in Section 3, and thus, this contribution to sample impurity was accounted for.

### 2.3 Viscosity

Viscosities were measured in parallel with the density measurements. A rolling ball viscometer, manufactured by Anton Paar, model DMA 4100, was used to measure the viscosities. The capillary diameter was 1.59 mm. The instrument self-optimizes the tilt angle. Like a density meter, it is important to have no air bubbles inside the capillary to obtain accurate data. The temperature accuracy of the viscometer was 0.02 K. At least three replicate measurements were conducted, and the reported viscosities are the averages. The relative standard deviation (RSD) was less than 0.01, typically about 0.005. The viscometer was calibrated with pure water. To check the validity of the calibration for higher viscosities, a sample of poly(ethylene glycol) (PEG200) was measured at higher temperatures, and the values agreed with published data^10^ within 1%. Further accuracy assessment is presented in Section 4.1, which includes a discussion of the viscosity measurement uncertainty.

### 2.4 Self-Diffusion Measurements

Self-diffusion measurements were obtained using a variable temperature broadband probe with a Bruker Avance 300 NMR spectrometer. The sample temperature was calibrated using the known temperature-dependent chemical shifts of ethylene glycol.^11^ Significant day-to-day variations of temperature were noted that limited the temperature uncertainty to 0.5 K. Each sample was given about 20 min time for temperature equilibration. The samples were not spun during data acquisition. The pulse sequence used for the self-diffusion measurements was based on a double stimulated echo pulse sequence using bipolar gradients and three spoiler gradients.^12,13^ Delays for eddy current recovery and gradient recovery were set at 5 and 0.2 ms, respectively. The relaxation delays varied from 3 to 6 s depending on the sample temperature. The gradient strength was varied linearly from 4.95 to 49.5 G·mm^–1^, resulting in 16 increments where the number of repetition and dummy scans was 16 and 4, respectively. The self-diffusion coefficients were obtained by fitting the obtained gradient dependence of the stimulated spin–echo intensity, *I*(*g*), for each of the proton signals according to eq 1.^14^
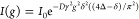
1where *I*_0_ is the reference spin–echo intensity in the absence of a gradient, γ is the ^1^H gyromagnetic ratio, Δ is the diffusion time (0.1 s), and *δ* is the length of the sine-shaped gradient pulse (varies for each sample and temperature condition). Depending on the identity of the sample, up to six signals, excluding the signal from the hydroxy proton, were observable. These provided independent measurements of the self-diffusion coefficient and are reported as the averages. Based on the obtained standard deviations and the temperature uncertainty as well as the sample uncertainty, the standard uncertainty of the self-diffusion coefficients is estimated to be 3 × 10^–11^ m^2^·s^–1^, which results in a relative standard uncertainty of up to 0.22 for the measured lowest self-diffusion coefficients.

## 3 Results

**Figure 1:**
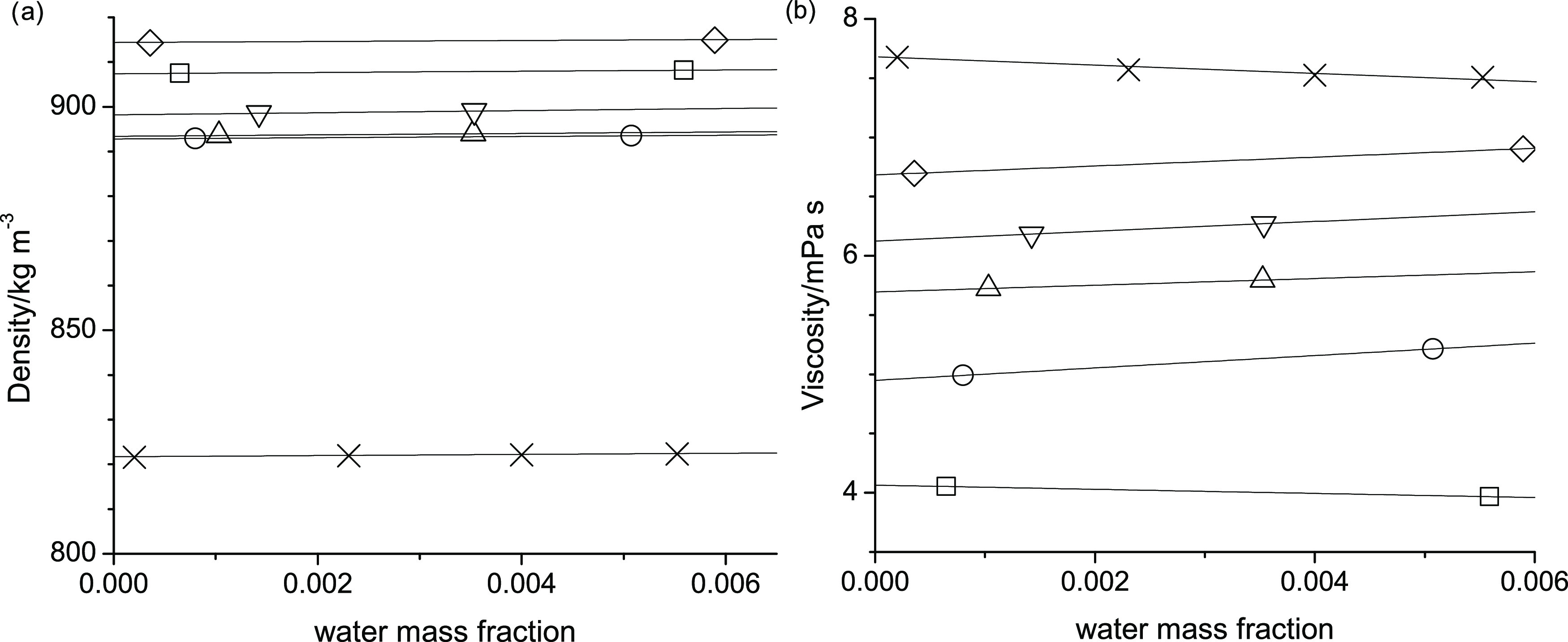
Density (a) and viscosity (b) as a function of water mass fraction for 2-pentoxyethan-1-ol (squares), 3-butoxypropan-1-ol (circle), 4-propoxybutan-1-ol (triangle-up), 5-ethoxypropan-1-ol (triangle-down), 6-methoxyhexan-1-ol (diamond), and octan-1-ol (cross). The lines are linear regressions that serve as a guide to the eye.

Tables 2, 3, 4, 5, 6, and 7 summarize all measured density, viscosity, and self-diffusion coefficients of octan-1-ol and the related ether-alcohols. Reported uncertainties for the density measurements are limited by the sample purity, and the uncertainties for viscosity and self-diffusion coefficients are limited by the instruments and random error, as further discussed in Section 4.1. The measured values reported in Tables 2–7 are organized by the water content in columns and by the temperature in rows. For convenience, the concentrations of water are presented as mole fractions as well as mass fractions, *w*, 10^–6^, as reported using the Karl Fischer titrator. As can be seen in Figure 1, the densities and viscosities of octan-1-ol vary linearly with the water content. Therefore, the intercepts of linear regressions represent the properties of dry octan-1-ol. The linear regression results reported in Tables 2–7 are based on using the mass fraction as the independent variable. The uncertainties of the self-diffusion coefficients are too high to observe these small changes with increasing water content, and Table 2 includes the average of the water content-dependent values. Due to the expense of the ether-alcohols, limited sample amounts allowed for only one water addition. Nevertheless, an extrapolation to zero water content is included in Tables 3–7 under the assumption that linear water dependencies for density and viscosity are also valid for the ether-alcohols.

**Table 2:** Density, Viscosity, and Self-Diffusion Coefficient of Octan-1-ol with Varying Water Content (Mass Fraction, *w*, as well as Mole Fraction, *x*_*w*_) at Ambient Pressure (0.10 ± 0.01 MPa)^a^

	*w*, 10^–6^					
	202	2305	4000	5525		
	*x*_*w*_					
*T*, K	0.00146	0.01642	0.02821	0.03860	slope	intercept
density, kg·m^–3^						
298.15	821.6	821.9	822.1	822.3	130.5 ± 35	821.6^b^
308.15	814.6	814.9	815.1	815.3	130.5 ± 35	814.6^b^
318.15	807.6	807.8	808.0	808.2	112.6 ± 40	807.6^b^
328.15	800.4	800.6	800.8	801.0	112.6 ± 59	800.4^b^
338.15	793.1	793.3	793.5	793.7	112.6 ± 17	793.1^b^
348.15	785.6	785.8	786.0	786.1	96.6 ± 63	785.6^b^
358.15	777.9	778.1	778.3	778.4	96.6 ± 63	777.9^b^
viscosity, mPa·s						
298.15	7.677	7.570	7.524	7.507	–32.2 ± 6.4	7.666 ± 0.023
308.15	5.466	5.395	5.366	5.364	–19.5 ± 5.2	5.456 ± 0.019
318.15	4.005	3.958	3.940	3.933	–13.6 ± 3.0	4.000 ± 0.011
328.15	3.008	2.977	2.969	2.960	–8.8 ± 1.8	3.005 ± 0.007
338.15	2.312	2.289	2.288	2.280	–5.6 ± 1.4	2.309 ± 0.005
348.15	1.813	1.798	1.798	1.789	–4.1 ± 0.9	1.812 ± 0.003
358.15	1.448	1.438	1.440	1.433	–2.5 ± 0.8	1.447 ± 0.003
self-diffusion coefficient, 10^–11^ m^2^ s^–1^						
299.2	14.06	14.37^c^	15.82	14.38	0	14.8 ± 0.9
309.2	20.66	17.94^c^	21.99	20.69	0	21.1 ± 0.8
319.0	28.10	21.56^c^	30.72	28.87	0	29.2 ± 1.3
328.6	37.63	26.06^c^	40.39	39.45	0	39.2 ± 1.4
338.3	49.79	32.34^c^	53.25	52.27	0	51.8 ± 1.8
347.7	63.16	38.00^c^	67.18	67.75	0	66.0 ± 2.5
357.0	79.83	76.94^c^	85.84	88.26	0	84.6 ± 4.3

a The relative standard uncertainty of *w* and *x*_*w*_ is 0.05. Temperature standard uncertainty is estimated to be 0.02 K for the density and viscosity measurements and 0.5 K for the self-diffusion coefficient measurements. The standard uncertainty of density, viscosity, and self-diffusion coefficient are 1.5 kg·m^–3^, 0.5 mPa·s, and 3 × 10^–11^ m^2^·s^–1^, respectively.

b The uncertainties of the intercepts for density are precise to the ten-thousandths place and thus are not listed.

c This entire set of self-diffusion data was omitted from the reported average because the Grubbs test confirmed all but the value at 298.15 K to be outliers.

**Table 3:** Density, Viscosity, and Self-Diffusion Coefficient of 2-Pentoxy-ethan-1-ol with Varying Water Content (Mass Fraction, *w*, as well as Mole Fraction, *x*_*w*_) at Ambient Pressure (0.10 ± 0.01 MPa)^a^

	*w*, 10^–6^			
	647	5589		
	*x*_*w*_			
*T*, K	0.00473	0.03961	slope	intercept
density, kg·m^–3^				
298.15	907.5	908.2	141.6	907.4
308.15	899.4	900.0	121.4	899.3
318.15	891.1	891.8	141.6	891.0
328.15	882.7	883.4	141.6	882.6
338.15	874.3	874.9	121.4	874.2
348.15	865.7	866.3	121.4	865.6
358.15	856.9	857.6	141.6	856.8
viscosity, mPa·s				
298.15	4.053	3.967	–17.4	4.064
308.15	3.067	3.010	–11.5	3.074
318.15	2.388	2.350	–7.7	2.394
328.15	1.906	1.878	–5.7	1.910
338.15	1.551	1.533	–3.6	1.553
348.15	1.285	1.272	–2.6	1.287
358.15	1.082	1.072	–2.0	1.083
self-diffusion coefficient, 10^–11^·m^2^·s^–1^				
299.2	29.7	29.0	0.0	29.4
309.2	40.4	38.1	0.0	39.3
319.0	51.8	50.5	0.0	51.2
328.6	66.7	63.7	0.0	65.2
338.3	82.5	79.4	0.0	81.0
347.7	101.2	94.0	0.0	97.6
357.0	122.1	117.0	0.0	119.5

a The relative standard uncertainty of *w* and *x*_*w*_ is 0.05. Temperature standard uncertainty is estimated to be 0.02 K for the density and viscosity measurements and 0.5 K for the Self-diffusion coefficient measurements. The standard uncertainty of density, viscosity, and self-diffusion coefficient are 4.5 kg·m^–3^, 0.5 mPa·s, and 3 × 10^–11^ m^2^·s^–1^, respectively.

**Table 4:** Density, Viscosity, and Self-Diffusion Coefficient of 3-Butoxypropan-1-ol with Varying Water Content (Mass Fraction, *w*, as well as Mole Fraction, *x*_*w*_) at Ambient Pressure (0.10 ± 0.01 MPa)^a^

	*w*, 10^–6^			
	800.6	5074.7		
	*x*_*w*_			
*T*, K	0.00585	0.03608	slope	intercept
density, kg·m^–3^				
298.15	892.9	893.5	140.4	892.8
308.15	885.0	885.7	163.8	884.9
318.15	877.1	877.7	140.4	877.0
328.15	869.1	869.7	140.4	869.0
338.15	860.9	861.5	140.4	860.8
348.15	852.7	853.2	117.0	852.6
358.15	844.3	844.8	117.0	844.2
viscosity, mPa·s				
298.15	4.990	5.214	52.4	4.949
308.15	3.713	3.859	34.2	3.686
318.15	2.845	2.942	22.7	2.827
328.15	2.234	2.301	15.7	2.221
338.15	1.792	1.839	11.0	1.783
348.15	1.463	1.497	8.0	1.457
358.15	1.214	1.239	5.8	1.209
self-diffusion coefficient, 10^–11^·m^2^·s^–1^				
299.2	22.4	22.1	0	22.3
309.2	30.9	30.0	0	30.5
319.0	40.6	39.8	0	40.2
325.4	52.6	51.1	0	51.9
338.3	65.8	64.6	0	65.2
347.7	81.3	79.6	0	80.5
357.0	101.5	97.4	0	99.5

a The relative standard uncertainty of *w* and *x*_*w*_ is 0.05. Temperature standard uncertainty is estimated to be 0.02 K for the density and viscosity measurements and 0.5 K for the Self-diffusion coefficient measurements. The standard uncertainty of density, viscosity, and self-diffusion coefficient are 0.6 kg·m^–3^, 0.5 mPa·s, and 3 × 10^–11^ m^2^·s^–1^, respectively.

**Table 5:** Density, Viscosity, and Self-Diffusion Coefficient of 4-Propoxybutan-1-ol with Varying Water Content (Mass Fraction, *w*, as well as Mole Fraction, *x*_*w*_) at Ambient Pressure (0.10 ± 0.01 MPa)^a^

	*w*, 10^–6^			
	1031.3	3526.7		
	*x*_*w*_			
*T*, K	0.00752	0.02588	slope	intercept
density, kg·m^–3^				
298.15	893.6	894.0	160.3	893.4
308.15	885.9	886.3	160.3	885.7
318.15	878.1	878.5	160.3	877.9
328.15	870.3	870.7	160.3	870.1
338.15	862.3	862.7	160.3	862.1
348.15	854.3	854.6	120.2	854.1
358.15	846.1	846.4	120.2	846.0
viscosity, mPa·s				
298.15	5.724	5.795	28.5	5.695
308.15	4.237	4.279	16.8	4.220
318.15	3.229	3.258	11.6	3.217
328.15	2.527	2.542	6.0	2.521
338.15	2.018	2.028	4.0	2.014
348.15	1.641	1.647	2.4	1.639
358.15	1.356	1.359	1.2	1.355
self-diffusion coefficient, 10^–11^·m^2^·s^–1^				
299.6	19.9	20.4	0.0	20.2
310.6	28.7	28.2	0.0	28.5
321.6	39.8	37.5	0.0	38.7
332.3	52.4	47.5	0.0	50.0
343.0	68.7	61.7	0.0	65.2
353.5	87.7	77.4	0.0	82.5
363.8	104.7	93.9	0.0	99.3

a The relative standard uncertainty of *w* and *x*_*w*_ is 0.05. Temperature standard uncertainty is estimated to be 0.02 K for the density, for viscosity measurements, and 0.5 K for the Self-diffusion coefficient measurements. The standard uncertainty of density, viscosity, and self-diffusion coefficient are 1.8 kg·m^–3^, 0.5 mPa·s, and 3 × 10^–11^ m^2^·s^–1^, respectively.

**Table 6:** Density, Viscosity, and Self-Diffusion Coefficient of 5-Ethoxypentan-1-ol with Varying Water Content (Mass Fraction, *w*, as well as Mole Fraction, *x*_*w*_) at Ambient Pressure (0.10 ± 0.01 MPa)^a^

	*w*, 10^–6^			
	1423.5	3536.1		
	*x*_*w*_			
*T*, K	0.01045	0.02595	slope	intercept
density, kg·m^–3^				
298.15	898.6	899.1	236.7	898.3
308.15	891.1	891.6	236.7	890.8
318.15	883.5	884.0	236.7	883.2
328.15	875.8	876.3	228.8	875.5
338.15	868.1	868.5	189.3	867.8
348.15	860.2	860.6	189.3	859.9
358.15	852.2	852.6	189.3	851.9
viscosity, mPa·s				
298.15	6.183	6.270	41.2	6.124
308.15	4.581	4.638	27.0	4.543
318.15	3.496	3.533	17.5	3.471
328.15	2.734	2.758	11.4	2.718
338.15	2.182	2.199	8.0	2.171
348.15	1.772	1.783	5.2	1.765
358.15	1.461	1.469	3.8	1.456
self-diffusion coefficient, 10^–11^·m^2^·s^–1^				
299.2	19.3	19.3	0.0	19.3
309.2	26.0	27.2	0.0	26.6
319.0	35.0	35.1	0.0	35.1
328.6	46.6	46.0	0.0	46.3
338.3	57.3	58.3	0.0	57.8
347.7	71.6	71.9	0.0	71.8
357.0	87.9	87.4	0.0	87.7

a The relative standard uncertainty of *w* and *x*_*w*_ is 0.05. Temperature standard uncertainty is estimated to be 0.02 K for the density, for viscosity measurements, and 0.5 K for the Self-diffusion coefficient measurements. The standard uncertainty of density, viscosity, and self-diffusion coefficient are 1.8 kg·m^–3^, 0.5 mPa·s, and 3 × 10^–11^ m^2^·s^–1^, respectively.

**Table 7:** Density, Viscosity, and Self-Diffusion Coefficient of 6-Methoxyhexan-1-ol with Varying Water Content (Mass Fraction, *w*, as well as Mole Fraction, *x*_*w*_) at Ambient Pressure (0.10 ± 0.01 MPa)^a^

	*w*, 10^–6^			
	357.3	5893.85		
	*x*_*w*_			
*T*, K	0.00262	0.04169	slope	intercept
density, kg·m^–3^				
298.15	914.3	914.9	108.4	914.3
308.15	906.9	907.5	108.4	906.9
318.15	899.4	900.1	126.4	899.4
328.15	891.9	892.5	108.4	891.9
338.15	884.3	884.8	90.3	884.3
348.15	876.5	877.0	90.3	876.5
358.15	868.6	869.1	90.3	868.6
viscosity, mPa·s				
298.15	6.696	6.904	37.6	6.683
308.15	4.943	5.065	22.0	4.935
318.15	3.760	3.835	13.5	3.755
328.15	2.934	2.979	8.1	2.931
338.15	2.337	2.365	5.1	2.335
348.15	1.896	1.913	3.1	1.894
358.15	1.562	1.572	1.8	1.561
self-diffusion coefficient, 10^–11^·m^2^·s^–1^				
299.2	18.2	18.5	0	18.3
309.2	25.3	26.2	0	25.7
319.0	32.9	33.5	0	33.2
325.4	42.2	43.3	0	42.8
338.3	55.6	56.6	0	56.1
347.7	70.6	71.4	0	71.0
357.0	85.2	83.1	0	84.2

a The relative standard uncertainty of *w* and *x*_*w*_ is 0.05. Temperature standard uncertainty is estimated to be 0.02 K for the density, for viscosity measurements, and 0.5 K for the Self-diffusion coefficient measurements. The standard uncertainty of density, viscosity, and self-diffusion coefficient are 4.5 kg·m^–3^, 0.5 mPa·s, and 3 × 10^–11^ m^2^·s^–1^, respectively.

There are several noteworthy trends in Figure 1 to point out. With respect to density (Figure 1a), its dependence on water content is small but consistently increasing for all six investigated alcohols. However, the viscosities in Figure 1b decrease with water addition for octan-1-ol and 2-pentoxyethan-1-ol but increase for the other ether-alcohols. The intercepts in Tables 2–7, i.e., the dry densities and viscosities, vary by less than 1% and between 1 and 3%, respectively, from the measurements obtained from the samples with the largest water content. These changes are small but noticeable within the reproducibility of the measurements.

**Figure 2:**
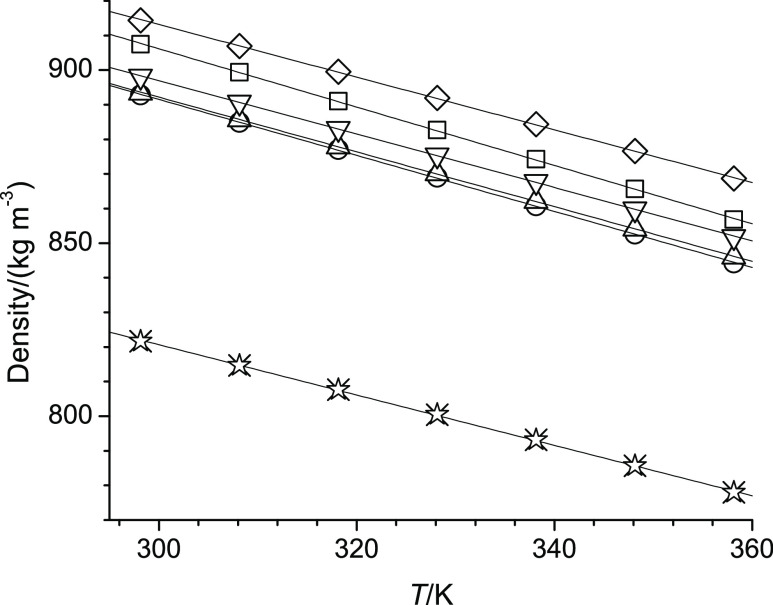
Temperature dependence of density for 2-pentoxyethan-1-ol (squares), 3-butoxypropan-1-ol (circles), 4-propoxybutan-1-ol (triangle-up), 5-ethoxypropan-1-ol (triangle-down), 6-methoxyhexan-1-ol (diamonds), and octan-1-ol (cross). Also shown are data from Fleshman et al.^15^ (stars) for octan-1-ol, which completely overlap the data reported here.

Figure 2 illustrates that the density decreases linearly with increasing temperature for all alcohols studied. The densities of octan-1-ol are about 75 kg·m^–3^ lower than the corresponding densities of the ether-alcohols. At 298.15 K, the densities of the ether-alcohols range from 890 to 920 kg·m^–3^. As can be seen from the parallel lines in Figure 2, the slopes are of nearly identical value for all of the studied alcohols.

Figure S1 shows the temperature dependencies of the molar volumes of the studied alcohols obtained from the intercept values of the densities, i.e., the densities of the dry alcohols, listed in Tables 2–7. These temperature dependencies are linear, as shown by the least linear regression lines in Figure S1. The linear temperature dependence leads to a ready evaluation of the thermal expansion coefficients, α, an industrially important materials property, using the defining eq 2
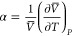
2where *T* is the temperature and the subscript *P* indicates constant pressure. The obtained values are listed in Table S1.

**Figure 3:**
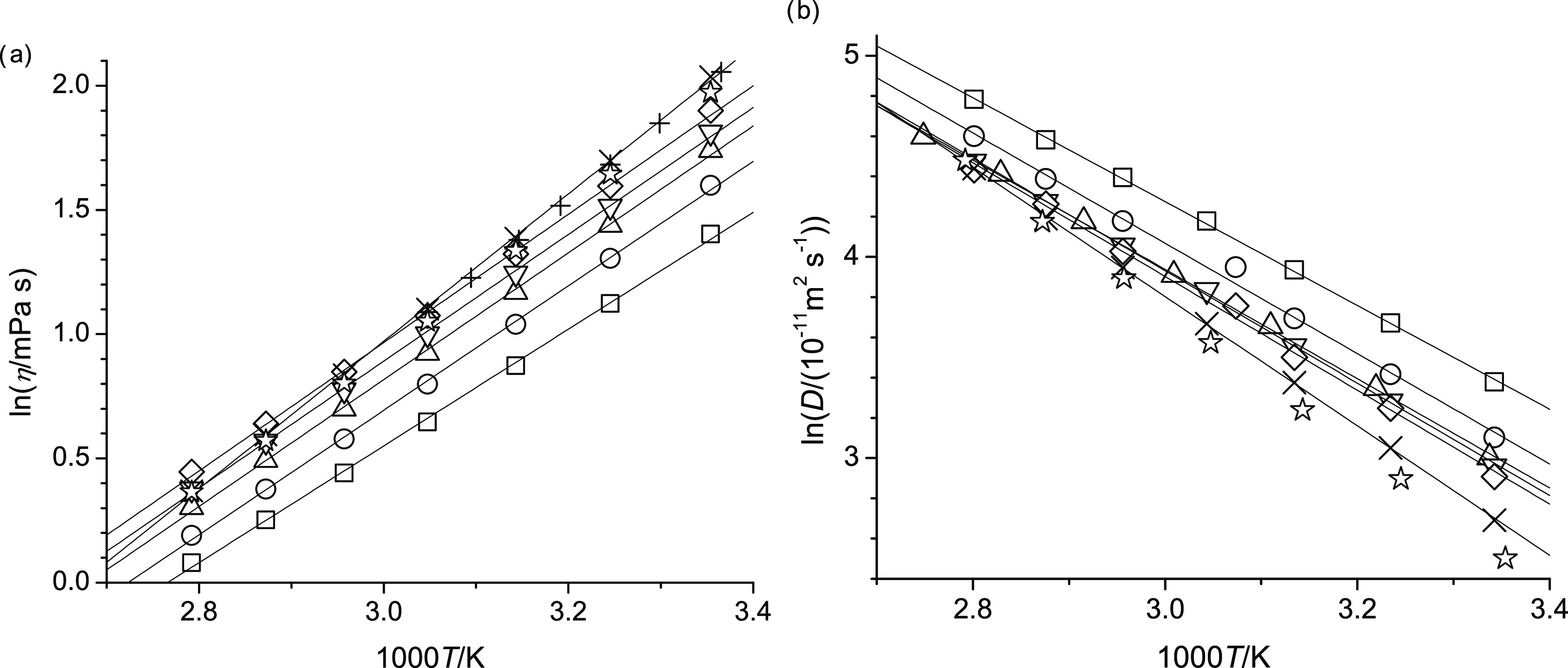
Arrhenius plots for viscosity (a) and self-diffusion coefficient (b) for 2-pentoxyethan-1-ol (squares), 3-butoxypropan-1-ol (circles), 4-propoxybutan-1-ol (triangles-up), 5-ethoxypropan-1-ol (triangles-down), 6-methoxyhexan-1-ol (diamonds), and octan-1-ol (cross). Viscosity data for octan-1-ol from Palombo et al.^16^ (plus) and Fleshman et al.^15^ (star) are included.

Figure 3a,b shows the ln of viscosity and self-diffusion coefficients as functions of inverse temperature, respectively, according to the logarithmic form of the Arrhenius equation, also referred to as the Arrhenius Guzmán equation with respect to viscosity measurements, shown in eq 3.
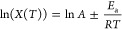
3

In eq 3, X(*T*) represents the temperature-dependent property, *R* is the universal gas constant, and *A* and *E*_a_ are the fit parameters known as the pre-exponential factor and activation energy, respectively. The sign before the second term of the right-hand equation indicates the increasing vs decreasing inverse temperature dependence for viscosity and self-diffusion coefficient, respectively.

In Figure 3, the data points for each alcohol follow the Arrhenius law well over the investigated range of temperatures, as can be seen by the added linear least-squares fits. The fit coefficients and their uncertainties are summarized in Table S2. Octan-1-ol has a larger slope magnitude relative to the ether-alcohols, which indicates an increased sensitivity to temperature for both viscosity and self-diffusion coefficients. The ether-alcohols all have very similar slope values, resulting in near-parallel lines in Figure 3.

The opposite temperature dependence of viscosity, η, and self-diffusion coefficient, *D*, in Figure 3 is readily understood from their inverse relationship as revealed in the Stokes–Einstein equation
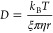
4where *k*_B_ is the Boltzmann constant, *ξ* is a dimensionless constant typically ranging between 4 and 6, and *r* is the hydrodynamic radius of the diffusing molecule. Further inspection of the Stokes–Einstein equation, as done in Section 4.3, requires values of *D* and *η* to be measured at the same temperature, which is generally not the case in Tables 2–7. For that reason, the Arrhenius fit parameters listed in Table S2 were applied to the self-diffusion data for interpolation.

## 4 Discussion

### 4.1 Data Quality

Given the sample impurities listed in Table 1, the relative measurement uncertainty contribution caused by these impurities ranges approximately between 0.005 to 0.6.^17^ For the density measurements, these relative uncertainties due to sample impurity are higher in value than relative uncertainties obtained for octan-1-ol in Table 2 that are based on the y-intercept uncertainty of the density dependence on water content. Hence, the density uncertainties in Tables 2–7 reflect the uncertainty of the unknown sample impurity. However, it appears that accuracy is much better than suggested by these uncertainties because the comparison of our density measurements with those reported in the literature, shown in Table 8, reveals excellent agreement, where none of the literature data deviates by more than 0.112% and agreement is mostly within 0.05%. In addition to the entries in Table 8, Figure 2 includes one data set by Fleshman et al.,^15^ which is also in excellent agreement with the data reported here as the data points completely overlap in Figure 2

**Table 8:** Percent Relative Deviation of Literature Densities and Viscosities of Octan-1-ol with Values from This Study

*T*, K	density						
298	0.004^25^	0.009^26^	0.022^27^	0.071^28^	–0.051^29^	–0.112^30^	–0.014^31^
	0.061^32^	–0.026^33^	0.014^34^	–0.014^35^	0.010^36^	–0.017^37^	0.022
	–0.009^38^	0.022^39^	–0.014^40^	0.000^35^	–0.008^41^	–0.112^42^	
308	0.006^30,42^	0.007^28,33,41^	0.008^27,38^				
318	0.014^27,28^	0.013^42^					

*T*, K	viscosity					
298.15	–3.9^27^	–4.0^28^	–2.8^29^	–6.8^31^	–2.2^33^	0.0^35^
308.15	–3.8^27^	–3.7^28^	–2.1^33^			
318.15	–3.4^27^	–3.3^28^				

The relative viscosity uncertainties for octan-1-ol in Table 2 that are based on the *y*-intercept uncertainty are comparable to the relative uncertainties from the sample impurities. However, a comparison with literature data in Table 8 shows that our measurements are consistently higher by up to 6.8%, except for one data point at 298.15 K, which agrees with our value. Not shown in Table 8 are two data sets, which are the only data sets we are aware of that cover a wide range of temperatures and are, for that reason, included in Figure 3. The data set by Palombo et al.^16^ agrees with our data by less than 1.5%, while the data set by Fleshman et al. is lower by about 5%. Overall, viscosity measurement uncertainty is not limited by sample impurity but by instrument uncertainty, which is estimated to be 0.5 mPa·s based on the deviations from most of the available literature values.

As for the self-diffusion measurements, we are aware of only one data set for octan-1-ol reported by Fleshman et al.^15^ that covers the temperature range of experimental measurements reported here and is included in Figure 3. Their reported values agree with our values within 3.7 × 10^–11^ m^2^·s^–1^, which is nearly within the estimated uncertainty of 3 × 10^–11^ m^2^·s^–1^ (see Section 2.4). In addition, McCall and Douglass report a value of 13.8 × 10^–11^ m^2^·s^–1^ at 298.15 K,^18^ while Cui et al. report a value of 14.1 × 10^–11^ m^2^·s^–1^ for the same temperature. These two values are within 1 × 10^–11^ m^2^·s^–1^ compared to the corresponding value reported in Table 2, which is less than the estimated uncertainty of 3 × 10^–11^ m^2^·s^–1^.

Finally, we are aware of two density measurements reported in the literature at 298.15 K for 2-(pentyloxy)-ethan-1-ol. Cooper and Partridge^19^ report a value of 949.9 kg·m^–3^, while Ashburn reports a value of 889.3 kg·m^–3^.^20^ In addition, there are several density measurements reported at lower temperatures: 892.7,^21^ 900.3,^22^ and 815.4 kg·m^–3^,^23^ at 293.15 K and 892.6 kg·m^–3^ at 288.15 K.^24^ These reported density values differ vastly, where the value of 900.3 kg·m^–3^ is the most recent reported value (1973) of these and agrees closest with the respective data entry of 907.4 kg·m^–3^ in Table 3.

### 4.2 Property Comparison Across Alcohols

**Figure 4:**
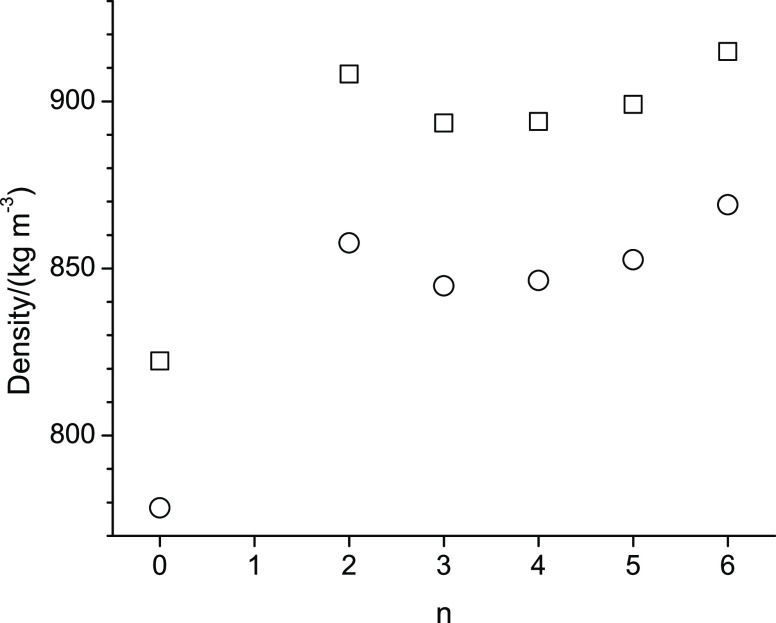
Densities at 298.15 K (square) and 358.15 K (circle) of octan-1-ol related ether-alcohols where n represents the CH_2_ group to which an alkoxy group is attached. The case of n = 0 represents octan-1-ol.

**Figure 5:**
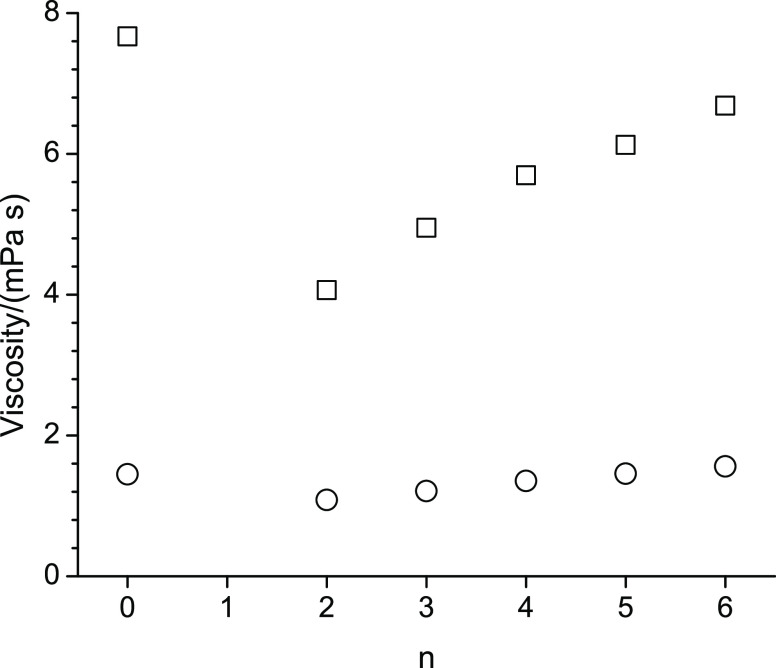
Viscosities at 298.15 K (square) and 358.15 K (circle) of octan-1-ol related ether-alcohols where n represents the CH_2_ group to which an alkoxy group is attached. The case of n = 0 represents octan-1-ol.

**Figure 6:**
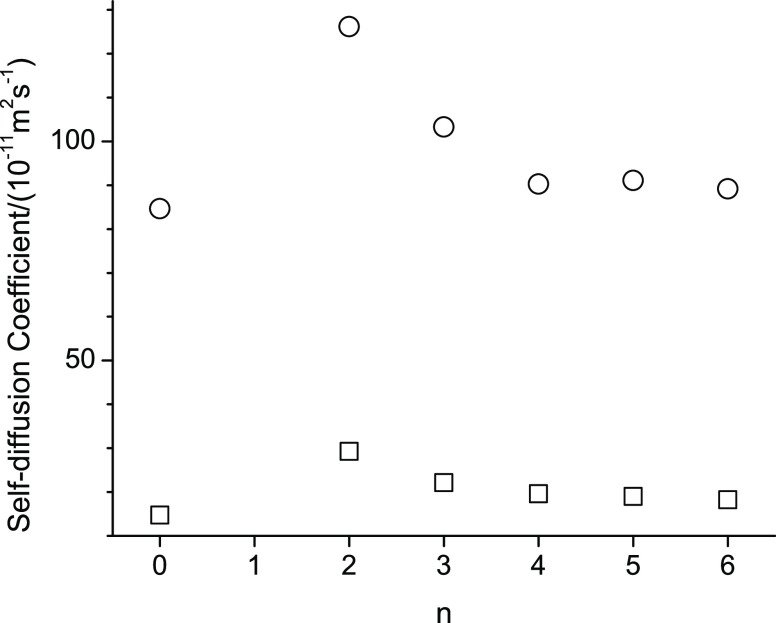
Self-diffusion coefficients at 298.15 K (square) and 358.15 K (circle) of octan-1-ol related ether-alcohols where n represents the CH_2_ group to which an alkoxy group is attached. The case of n = 0 represents octan-1-ol.

For easier comparison of the measured properties of the studied alcohols, Figures 4–6 show, respectively, for the lowest (298.15 K) and highest temperature (358.15 K), the dependence of the density, viscosity, and self-diffusion measurements with respect to the position of the ether function within the molecular structure of the ether alcohol. Specifically, in Figures 4–6, n represents the CH_2_ group to which the alkoxy group is attached. (The value n = 0 is assigned for octan-1-ol.)

**Figure 7:**
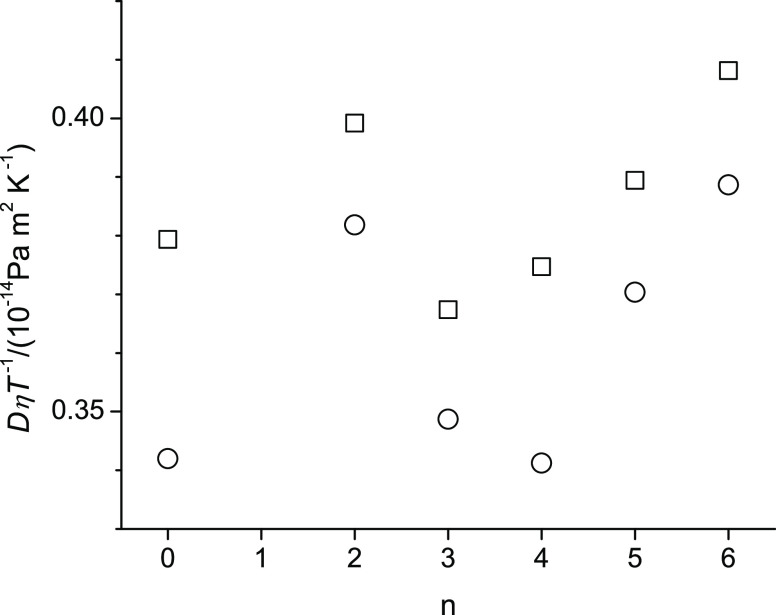
Product of Self-diffusion coefficient and viscosity at 298.15 K (square) and 358.15 K (circle) of octan-1-ol related ether-alcohols where n represents the CH_2_ group to which an alkoxy group is attached. The case of n = 0 represents octan-1-ol. The scale spans 0.09 units.

**Figure 8:**
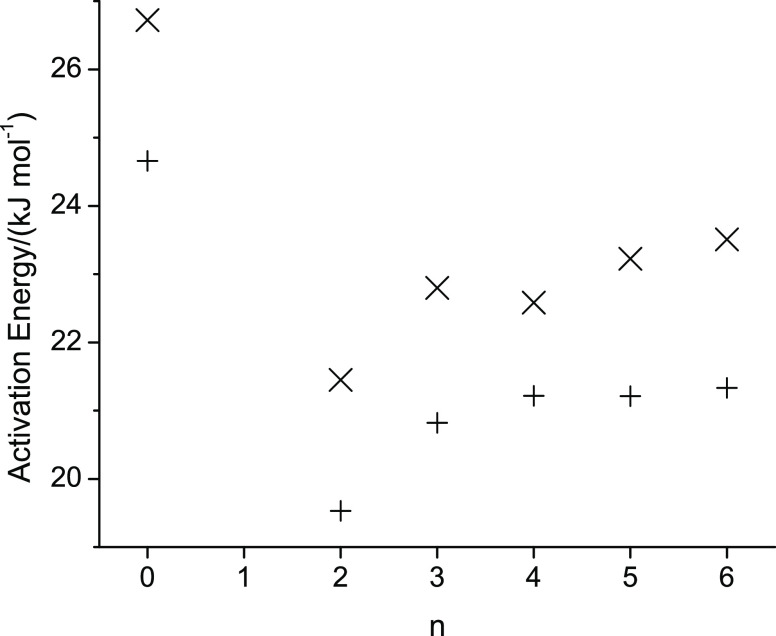
Activation energies for translational motion (cross) and momentum transfer (plus) of octan-1-ol related ether-alcohols where n represents the CH_2_ group to which an alkoxy group is attached. The case of n = 0 represents octan-1-ol.

Figures 4–8 display peculiar trends with respect to n. In Figure 4, the density increases from octan-1-ol to 2-pentoxyethan-1-ol (n = 2) by about 80 kg·m^–3^. As the n further increases, the densities in Figure 4 display a parabolic pattern with a minimum at n = 3. In Figure 5, the viscosity decreases from octan-1-ol to 2-pentoxyethan-1-ol (n = 2) and then increases in an approximately linear fashion from n = 2 to n = 6 at both temperatures. Comparing Figure 6 for the self-diffusion coefficient with Figure 5 for the viscosities, the shape of the n-dependence in Figure 6 is sort of a mirror image (inverted trends) of Figure 5, where the self-diffusion coefficients decrease (and not increase) from n = 2 to n = 6. This reverse n-dependence can be readily explained with the Stokes–Einstein equation, eq 3, where one can see that *D* ∼ 1/*η*. However, the self-diffusion coefficients decrease from n = 2 to n = 6 in a less linear fashion than the corresponding viscosity increase in Figure 4. A closer look into this matter is shown in Figure 7, where *DηT*^–1^ is plotted against n. According to eq 3, the n-dependence of *DηT*^–1^ is expected to be flat because the hydrodynamic radius should be the same for all alcohols given that the ether alcohols are all constitutional isomers with an identical molar weight that is nearly the same as that of octan-1-ol. This is not observed in Figure 7. Instead, the pattern of the n-dependence of *DηT*^–1^ is reminiscent to the n-dependence pattern of the density in Figure 4. Moreover, the data points in Figure 7 display a temperature dependence because the values at 358.15 K are all consistently lower than the corresponding values at 298.15 K. This is unexpected because the temperature dependence should have been removed by the division of *T* in *DηT*^–1^. Finally, Figure 8 shows the activation energies associated with the viscosities and self-diffusion measurements, each as a function of n. The largest activation energy is observed for octan-1-ol. The shapes of the graphs in Figure 8 resemble that of Figure 5 for viscosity, with a flatter dependence on n as n approaches n = 6. The activation energies for translational motion are consistently higher than the activation energies obtained from the viscosities, which represent the barriers to momentum transfer.

The trends described in Figures 4–8 can be explained in terms of the increased hydrogen bonding possibilities introduced by the ether functional group. Specifically, for octan-1-ol, hydrogen bonding can only occur intermolecularly between the hydroxy groups. The addition of the ether functional group offers intermolecular hydrogen bonding between hydroxy proton and ether oxygen. However, the presence of the ether functionality also brings about the possibility of intramolecular hydrogen bonding. The fact that the densities of all ether-alcohols are significantly higher compared to octan-1-ol suggests that the intermolecular hydrogen bonding interactions are increased based on the general concept that increased attractive interactions lead to a decreased demand in volume. However, intramolecular hydrogen bonds result in circular structure formations that may require more volume and thus cause a decrease in density. Ring formation is especially favorable for 6-member rings,^43^ which would correspond to n = 3, where indeed the density is lowest among the ether alcohols in Figure 4.

Interestingly, the increased hydrogen bonding interactions of the ether alcohols compared to octan-1-ol are also revealed in the *DηT*^–1^ graphs of Figure 7 because of the cancellation of the dynamics effects on self-diffusion (increased) and momentum transfer related to viscosity (decreased). Moreover, increased temperatures provide more kinetic energy to overcome hydrogen bonding interactions. A loss of intermolecular hydrogen bonds at higher temperatures may allow for a shift toward more intramolecular hydrogen bonding. This would explain the decreased *DηT*^–1^ values at higher temperatures in Figure 7.

Increased intermolecular hydrogen bonding interactions should require more energy to break these. The largest activation energy observed for octan-1-ol is thus in apparent disagreement with the observed increased densities. One possible explanation to rectify these diverging observations could lie in the increased diversity of intermolecular interactions, where the intermolecular hydrogen bonding interactions between hydroxy and ether molecules are weaker than the hydroxy–hydroxy intermolecular hydrogen bonding interactions. This would allow the molecules to disengage more easily from the hydrogen bonding interactions to jump into another solvation cage. The observation in Figure 8 that 2-pentoxyethan-1-ol (n = 2) displays the lowest activation energies in Figure 8 suggests that intermolecular hydrogen bonding is lower for this ether alcohol compared to other ether alcohols. One possible explanation is that the ether group is closest to the hydroxy group in 2-pentoxyethan-1-ol compared to the other ether alcohols. The closeness of the ether oxygen to the hydroxy group might lead to larger fluctuations of making and breaking hydrogen bonds between hydroxy–hydroxy and hydroxy-ether moieties, leading to an increase in the dynamics with concomitant reduction in the activation energies observed in Figure 8.

It is interesting that the activation energies in Figure 8 are consistently lower for momentum transfer (viscosity) compared to translational motion (self-diffusion), including for octan-1-ol. Apparently, the energy barrier to jump from a solvent cage to a solvent cage is for all alcohols higher than the barrier to transfer momentum from molecule to molecule. This suggests that the intermolecular hydrogen bonding interactions are very dynamic in nature. Breaking and reforming hydrogen bonds would allow for rotational movements while still preventing translational motions, thus leading to a higher activation energy for translational motion.

Next, we will discuss the diverging trends of water addition on density and viscosity in Figure 1. Water is a strong hydrogen bond acceptor and donor. Its density is higher than that of any of the alcohols studied here. Therefore, the small linear increase in density with respect to the water mass fraction in Figure 1a is readily explained by increased hydrogen bonding interactions from the introduced water. However, as for the viscosity in Figure 1b, the effect of water is more complicated. The viscosity of pure water is about 1 mPa·s at 298.15 K,^44^ which is significantly lower than the viscosity of octan-1-ol as well as of the ether-alcohols. A reduction of viscosity with the addition of water is therefore expected but is observed only for octan-1-ol and 2-pentoxyethan-1-ol. Interestingly, octan-1-ol is incapable of intramolecular hydrogen bonding, and intramolecular hydrogen bonding might not be favored for 2-pentoxyethan-1-ol due to the resulting ring constraints. The added water competes with the inter- and intramolecular alcohol hydrogen bonds, where conceivably, the intramolecular hydrogen bonds may be easier to break. Such reduction in intramolecular hydrogen bonding would in turn lead to increases in intermolecular interactions as the ether-alcohol may take on a more stretched molecular conformation, which would thus explain the increase in viscosities upon water addition observed in Figure 1b. We caution, however, that only two data points are present in Figure 2b per ether-alcohol and that the viscosity changes upon the addition of water are comparable to the estimated uncertainties in viscosity measurements.

### 4.3 Stokes–Einstein Relation

**Figure 9:**
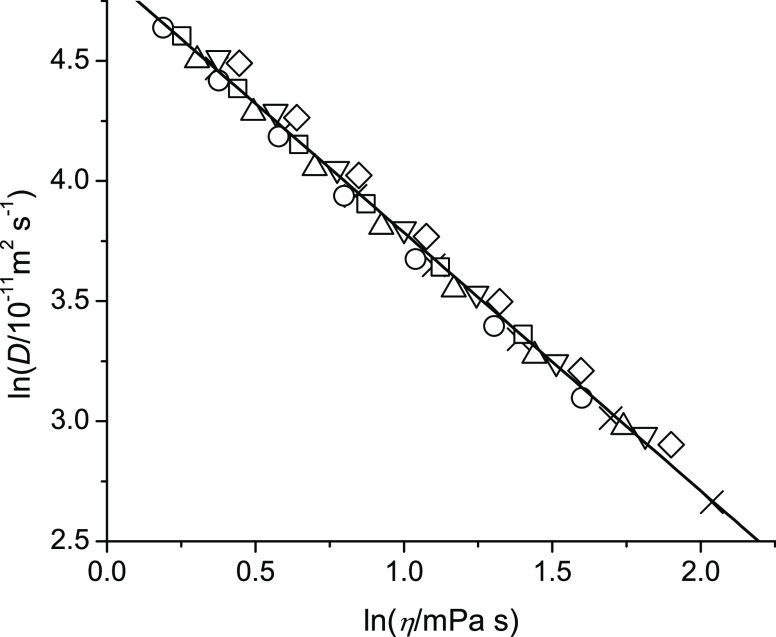
Natural log of self-diffusion plotted as a function of the natural log of viscosity at 298. 15 K of 2-pentoxyethan-1-ol (squares), 3-butoxypropan-1-ol (circle), 4-propoxybutan-1-ol (triangle-up), 5-ethoxypropan-1-ol (triangle-down), 6-methoxyhexan-1-ol (diamond), and octan-1-ol (cross). The combined data is fitted to a universal least-squares linear equation of *y* = −1.0755*x* + 4.86048 (solid line).

This section discusses the possibility that intermolecular hydrogen bonding interactions are strong enough to cause the formation of dimers or possibly aggregates. This would result in effective hydrodynamic radii that are larger than those for a single molecule. To investigate this matter, we utilize the Stokes–Einstein relation shown in eq 4, rearranged to calculate values for ξ. The values for ξ are expected to be between 4 for conditions where there are no interactions between the self-diffusing particles (the so-called slip boundary) and 6 for conditions where these interactions are strong (the stick boundary).^45^ The needed van der Waals radii were calculated with the method by Bondi^46^ as further detailed by Edward,^47^ resulting in values of 0.3322 nm for octan-1-ol and 0.3242 nm for the ether-alcohols. The obtained values for ξ are listed in Table S3 and range between 3.3 and 3.9, with larger values at higher temperatures. These values are all below the range of 4–6. However, the shown estimated uncertainties based on error propagation range from 0.5 to 1.6. It is also possible that the obtained radii may have been slightly overestimated, as was also recently observed for PEG200.^48^ Regardless, the formation of dimers or oligomers is not indicated because the hydrodynamic radius would significantly increase. To further confirm this conclusion, Figure 9 shows a double logarithmic plot of self-diffusion coefficients vs viscosity. If there were significant dimer or oligomer formations for these alcohols, then the extent of such dimer or oligomer formation would likely differ between the molecules. However, as can be seen in Figure 8, the data from all studied alcohols can be fitted to one universal linear fit equation, as indicated in the caption of Figure 9. The slope of −1.0755 is in magnitude higher than the expected value of −1 based on eq 4, which could be a reflection of the lower activation energies for momentum transfer (viscosity) compared to translational motion (self-diffusion) observed in Figure 8.

## 5 Conclusions

New data of density, viscosity, and self-diffusion coefficients were presented for octan-1-ol and related ether-alcohol covering temperature ranges from 298.15 to 359.15 K. The effect of water impurity on these properties was found to be small but noticeable for density and viscosity. Densities increase for all alcohols upon the addition of water. Viscosities increase as well, except for octan-1-ol and 2-pentoxyethan-1-ol. Densities and molar volumes increase linearly with temperature, while the temperature dependencies of the viscosities and self-diffusion coefficients follow Arrhenius’ law for all alcohols over the investigated temperature range. The comparison of the properties across the different alcohols provided interesting insights into the effect of increased hydrogen bonding, as well as the introduction of intramolecular hydrogen bonding caused by the presence of the ether functionality in the ether alcohols. The increased hydrogen bonding causes an increase in density, which, however, is modulated to lower values for those ether-alcohols more likely to engage in intramolecular hydrogen bonding. There appears to be a differentiation between the underlying dynamics for translational motion and momentum transfer for all of the investigated alcohols based on the observation that the activation energies for translational motion are consistently higher than for momentum transfer. These differences cancel out for the mathematical product of self-diffusion coefficient and viscosity (*Dη*), which shows a similar trend across the ether-alcohols as the density. This further shows that density is governed by the energetics of the hydrogen bonding interactions, while self-diffusion and viscosity are governed by molecular dynamics. With respect to energetics, it appears that hydrogen bonding is strongest for intermolecular hydroxy–hydroxy hydrogen bonding compared to inter- and intramolecular hydroxy-ether hydrogen bonding. These hypothesized trends need further confirmation by theoretical studies. For that reason, MD simulations of these alcohols are underway and will be reported in due course.

## Supporting Information Available

Graph of molar volumes and tables of thermal expansion coefficients and Arrhenius fit parameters and values of the constant ξ of the Stokes–Einstein equation, eq 3. (PDF)
