# Supplementary material for: Densities, Viscosities, and Self-Diffusion Coefficients of Octan-1-ol and Related Ether-Alcohols
Source: J Chem Eng Data. 2024 Jul 3;69(8):2688–99. doi: 10.1021/acs.jced.4c00195 (PMC11317982; doi:10.1021/acs.jced.4c00195)
Supplement: Supplementary file 1 — je4c00195_si_001.pdf [file je4c00195_si_001.pdf]

Supporting Information

Densities, Viscosities, and Self-Diffusion Coefficients of octan-1-ol and Related Ether-Alcohols

Markus M. Hoffmann,*[1] Anthony A. Gonzalez,[1] Mandy T. Huynh,[1] Kashane K. Miller,[1] Torsten Gutmann,[2] and Gerd Buntkowsky*[2]

[1] *Department of Chemistry and Biochemistry, State University of New York Brockport, Brockport, NY, 14420, USA*

[2] *Institute of Physical Chemistry, Technical University Darmstadt, Alarich-Weiss-Straße 8, D-64287 Darmstadt, Germany*

Corresponding authors: mhoffman@brockport.edu
gerd.buntkowsky@chemie.tu-darmstadt.de

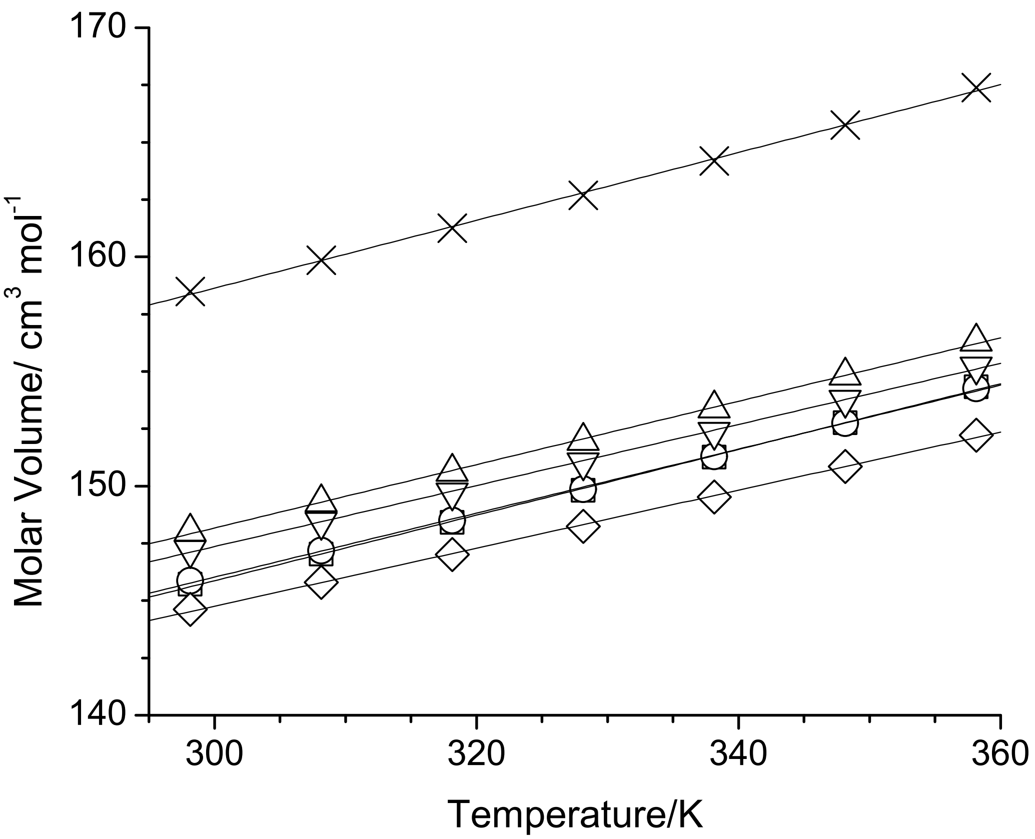

Figure S1. Molar volume as a function of temperature for 2-pentoxyethan-1-ol (squares), 3-butoxypropan-1-ol (circle), 4-propoxybutan1-1-ol (triangle-up), 5-ethoxypropan-1-ol (triangle-down), 6-methoxyhexan-1-ol (diamond), octan-1-ol (cross.)

Table S1. Thermal Expansion Coefficient, $\alpha/10^{-3}K^{-1}$, of octan-1-ol and related ether-alcohols at ambient pressure (0.10 ± 0.01 MPa)[a]

T/K	0[a]	2	3	4	5	6
298.15	0.9341	0.9834	0.9577	0.9343	0.9055	0.8755
308.15	0.9261	0.9747	0.9492	0.9262	0.8980	0.8684
318.15	0.9181	0.9657	0.9408	0.9181	0.8903	0.8613
328.15	0.9100	0.9566	0.9322	0.9099	0.8826	0.8541
338.15	0.9017	0.9475	0.9234	0.9015	0.8748	0.8468
348.15	0.9341	0.9834	0.9577	0.9343	0.9055	0.8755
358.15	0.9261	0.9747	0.9492	0.9262	0.8980	0.8684

[a] Standard uncertainties are estimated to be 0.02 K for *T* and 2*10^{-6} K^{-1} for α.

[b] number reflects the name, e.g., 2 = 2-pentoxyethan-1-ol, zero is for octan-1-ol

Table S2. Fit parameters to the Arrhenius equation for octan-1-ol (n = 0) and related ether-alcohols, where n reflects the name, e.g., 2 = 2-pentoxyethan-1-ol

	Viscosity		Self-diffusion Coefficients	
n	E_a/kJ·mol^{-1}	ln(A/mPa·s)	E_a/kJ·mol^{-1}	ln(A/10^{-11}m^2·s^{-1})
0	24.66 ± 0.21	-7.93 ± 0.08	26.72 ± 0.12	13.44 ± 0.05
2	19.53 ± 0.27	-6.50 ± 0.10	21.45 ± 0.20	12.01 ± 0.07
3	20.82 ± 0.25	-6.82 ± 0.09	22.80 ± 0.71	12.29 ± 0.26
4	21.22 ± 0.25	-6.84 ± 0.09	22.58 ± 0.26	12.09 ± 0.10
5	21.21 ± 0.22	-6.76 ± 0.08	23.23 ± 0.26	12.31 ± 0.09
6	21.33 ± 0.24	-6.78 ± 0.09	23.51 ± 0.60	12.39 ± 0.22

Table S3: Evaluation of the constant ξ of the Stokes Einstein Equation, eq 3, for octan-1-ol (n = 0) and related ether-alcohols, where n reflects the name, e.g., 2 = 2-pentoxyethan-1-ol.

T/K\n	0	2	3	4	5	6
298.15	3.5 ± 0.7	3.4 ± 0.5	3.7 ± 0.6	3.6 ± 0.6	3.5 ± 0.6	3.3 ± 0.6
308.15	3.5 ± 0.6	3.5 ± 0.6	3.8 ± 0.6	3.8 ± 0.6	3.6 ± 0.6	3.4 ± 0.5
318.15	3.6 ± 0.6	3.6 ± 0.8	3.9 ± 0.7	3.9 ± 0.7	3.6 ± 0.6	3.5 ± 0.6
328.15	3.7 ± 0.7	3.6 ± 1.0	3.9 ± 0.9	3.9 ± 0.8	3.7 ± 0.7	3.5 ± 0.6
338.15	3.7 ± 0.8	3.6 ± 1.2	3.9 ± 1.1	3.9 ± 1.0	3.7 ± 0.9	3.5 ± 0.8
348.15	3.8 ± 1.1	3.6 ± 1.4	3.9 ± 1.3	4.0 ± 1.2	3.7 ± 1.1	3.5 ± 0.9
358.15	3.9 ± 1.3	3.6 ± 1.6	3.9 ± 1.6	4.0 ± 1.5	3.7 ± 1.3	3.5 ± 1.1
